# Call me maybe: Risk factors of impaired social contact during the COVID‐19 pandemic and associations with well‐being

**DOI:** 10.1111/bjso.12546

**Published:** 2022-05-26

**Authors:** Selma C. Rudert, Stefan Janke

**Affiliations:** ^1^ University of Koblenz and Landau Landau Germany; ^2^ University of Mannheim Mannheim Germany

**Keywords:** anxiety, COVID‐19, depression, psychological needs, relatedness, self‐determination theory, social contact, well‐being

## Abstract

The COVID‐19 pandemic caused major societal changes worldwide, with the most notable being lockdowns and restrictions on social contact. We conducted a longitudinal study (total *n* = 1907) in Germany with two time points to (1) identify demographic risk factors of impaired social contact during the pandemic, as well as investigate potential consequences of (2) impaired social contact and (3) different modes of communication on individuals' well‐being during the first lockdown in spring 2020. Results indicate that particularly individuals living alone and being unable to work reported a lower frequency of (face‐to‐face) contact in comparison with participants living with others or working. Impaired social contact was indirectly associated with a negative development in well‐being (life satisfaction, anxiety and depression) over time, and this relation was mediated via relatedness. Moreover, the frequency of face‐to‐face and phone communication during lockdown was positively associated with relatedness and well‐being; however, digital communication was not. The findings stress the importance of maintaining social contact in times of social distancing and of fostering reconnection between individuals once the pandemic is over.

## BACKGROUND

In 2020, the COVID‐19 pandemic caused societies worldwide to implement a broad range of measures to slow down the spread of the virus. Among those were so‐called social distancing measures that aimed to reduce direct, physical contact between individuals. For instance, governments restricted public and private social gatherings, confined social interactions to clearly defined physical distances and members of one's own household, or even admitted curfews and restricted public movement (Hale et al., 2021). Consequently, the daily lives of many individuals changed practically overnight. Meeting friends or family, engaging in social activities or celebrating together were no longer allowed. In addition, many people found themselves working or studying from home instead of in workplaces, universities or schools.

From the very beginning, clinical psychologists as well as health and welfare organizations have issued concerns about negative effects of those limitations in social contact for people's psychological well‐being. These warnings often addressed negative long‐term side effects such as increased feelings of loneliness (Banerjee & Rai, 2020) and impaired mental health (Vahratian et al., 2021). Research has since focussed on *whether* individuals' psychological well‐being was impaired in the wake of the COVID‐19 health crisis (e.g. Prati & Mancini, 2021; Vindegaard & Benros, 2020; Xiong et al., 2020), with several studies indicating an increase in ill‐being (Brodeur et al., 2021, Zacher & Rudolph, 2021), depression (Ettman et al., 2020; Salari et al., 2020) and anxiety (Bäuerle et al., 2020).

However, several questions remain unanswered, namely *who* was mostly affected from the restrictions, through which *psychological mechanisms* reductions of social contact may have affected psychological well‐being and whether the *mode of communication* with others during lockdown affected psychological well‐being. The present contribution thus aims to answer three distinct research objectives: (1) We aim to identify demographic *risk factors* for a reduction in social contact during the first weeks of the lockdown measures within Germany in March/April 2020. Moreover, we aim to explain how (2) a reduction in *quantity of social contact* and (3) different *mode of communication* is associated with changes in psychological well‐being over time.

## SOCIAL CONTACT IN TIMES OF SOCIAL DISTANCING

**FIGURE 1:**
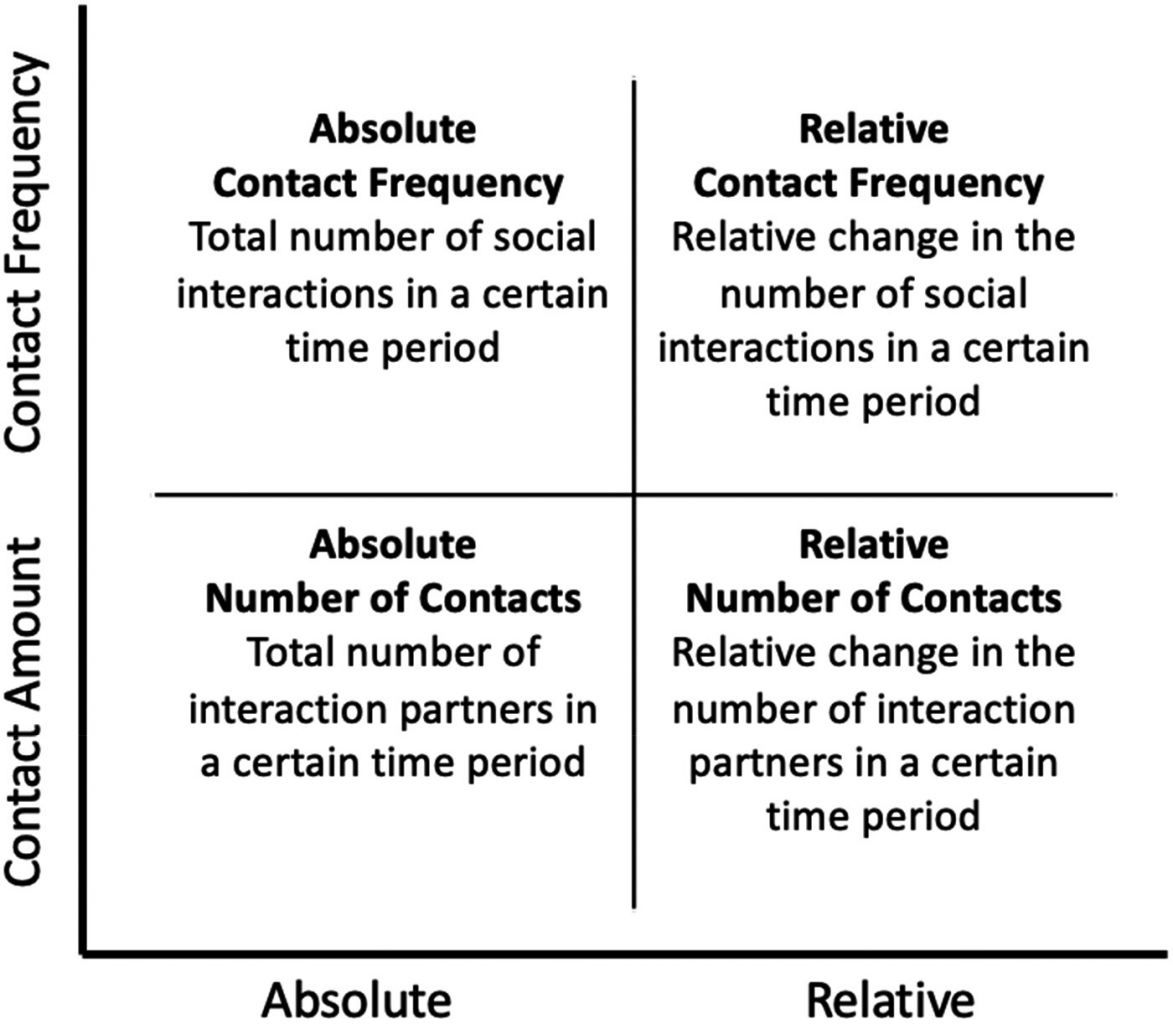
Multifaceted conceptualization of social contact quantity

As a first step towards investigating risk factors and consequences of (reduced) social contact, it is advantageous to understand social contact as a multifaceted construct. As defining characteristics of social interactions that were most likely to be affected by the lockdowns, we distinguish between the quantity of one's social contact (Fiorillo & Sabatini, 2011) and the mode of communication (Baym et al., 2004). The *quantity of social contact* describes the amount of interactions with other people. Quantity can be further distinguished in terms of *frequency* of social contact and the *number* of different individuals a person had contact with. Both factors may work in tandem, but are not necessarily identical: For instance, a couple living together might have a high contact frequency during lockdown, because they are interacting with their partner several times a day. At the same time, they might have a low number of contacts if they are interacting with no one else. We can further define social contact during the pandemic either by *absolute* numbers or by temporal comparison reflecting *relative change* in social contact during vs. before the lockdown. A perspective on relative change provides more context to the meaning of absolute numbers in terms of a *relative deprivation logic* (Walker & Smith, 2002): Especially for individuals who had only a few social contacts before the lockdown or mainly interacted with members of their own household, contact restrictions might not have resulted in substantial changes to their social life. In contrast, for individuals with large social networks who used to interact with many different people each day, contact restrictions might represent a massive change (Heidinger & Richter, 2020; Lee et al., 2020). In sum, we conceptualize the quantity of social contact in terms of a multifaceted model with *number* vs. *frequency of contacts* and *absolute* vs. *relative* as central axes (see Figure 1).

Besides the quantity of social contact, the pandemic likely contributed to a change in the *mode of communication*. The social distancing measures included a strong discouragement of direct, face‐to‐face contact. To compensate, politicians and media outlets encouraged the population to engage more frequently in non‐direct social contact, for instance via phone or digitally. This raises the question whether social contact via communication devices can compensate at least partly for a reduced frequency of face‐to‐face contact.

### Research objective 1: Risk factors for reduced social contact during the pandemic

The social distancing measures during the pandemic likely resulted in substantial changes both regarding the quantity of social contact and the mode of communication. Nevertheless, these changes may vary in strength for different groups within the population (e.g. Rudert et al., 2021). We thus investigate three demographic factors that could be associated with decreased social contact and shifts in mode of communication: age, living conditions and working environment.

First, *older age* might represent a risk factor. While not necessarily a risk factor for less social contact and loneliness per se (Prati & Mancini, 2021), some studies show a stronger *increase* in loneliness during lockdown or compared with pre‐lockdown data for older compared with younger adults (e.g. Luchetti et al., 2020, Buecker et al., 2020; albeit some studies found different results, see Rudert et al., 2021, for a review). A possible reason could be differences in the mode of communication: As younger individuals are typically more familiar with digital technology and communication than older adults (Hunsaker & Hargittai, 2018), it might be easier for them to compensate digitally for deficits in face‐to‐face social interactions.

Second, *living alone* might represent another risk factor (Heidinger & Richter, 2020). The social life of individuals who live alone was strongly limited in some countries (e.g. in Germany, individuals could only meet with one other person outside of one's own household). These individuals likely experienced a lower (relative) frequency and/or number of social contacts (particularly for face‐to‐face communication) compared to individuals living with others.

Third, the workplace represents an important source for a variety of social interactions. With the onset of the pandemic, many individuals were either obliged to shift their daily work from their normal place of occupation to their home office or could not work at all due to the pandemic, as governments issued executive orders that required firms and businesses to shut down on‐site work (Kramer & Kramer, 2020). However, some key industries were allowed to maintain on‐site workplaces (e.g. supermarkets, food industry and health care). We assume that individuals working from home or individuals who could not work at all due to the pandemic would experience a lower (relative) frequency and/or number of social contacts as well as less face‐to‐face communication compared with individuals working on‐site.

### Research objective 2: Consequences of reduced social contact during the pandemic

In addition to identifying risk factors, it is crucial to understand why reductions in social contact during lockdown might have negative consequences for psychological well‐being. Self‐Determination Theory (SDT; Ryan & Deci, 2017) posits that all humans share central basic psychological needs that are fundamental for psychological functioning (Ryan & Deci, 2017). One is the *need for relatedness*, describing both a desire for interpersonal and stable affective bonds, but also for frequent personal contact and interaction with close persons (Baumeister & Leary, 1995). Restrictions of social contact during the COVID‐19 pandemic are likely to impose immediate stress, as individuals may feel that they cannot effectively relate with close others, leaving the human urge for genuine and deep connection thwarted. In line with this assumption, several studies have empirically observed an increase in experienced loneliness during lockdown (see Rudert et al., 2021, for an overview).

An enforced reduction of social contact might also be negatively associated with two other basic psychological needs proclaimed by SDT: Contact restrictions may result in a thwarted *need for autonomy* (i.e. experience of volition and self‐direction in thought, feeling and action; Legault, 2016), as statewide measures impose external regulations on individuals that strongly restrict personal freedom (Cantarero et al., 2021). Additionally, loss of social contact might result in a thwarted *need for competence* (i.e. experience of the ability to act effectively; Legault, 2017) by limiting social resources that individuals deem necessary to cope with current challenges and master their daily life (see Vermote et al., 2022, for an extended discussion of basic psychological needs during the pandemic).

Experiencing episodes of decreased need satisfaction can impair life satisfaction (Walker & Kono, 2018) and foster ill‐being (Vansteenkiste & Ryan, 2013). Moreover, individuals who perceive their needs to be threatened typically respond with strong distress and anxiety, because need threatening situations severely limit the individual's ability to fulfil inner strivings for self‐actualization (Quested et al., 2011; Ryan & Deci, 2000). Impaired need satisfaction has also been identified as a key factor in the development of depressive symptoms (e.g. Campbell et al., 2018; Stenling et al., 2020). Given the centrality of need satisfaction for well‐being, it can be expected that factors that affect need satisfaction—such as presumably quantity and mode of social contact—should also be indirectly associated with impaired well‐being.

### Research objective 3: Consequences of a shift in the mode of communication during the pandemic

While face‐to‐face contact was prohibited during lockdown, authorities encouraged to stay connected via phone or digital technologies, a measure that has been highly debated: On the one hand, prior research indicates that alternative modes of social communication cannot sufficiently compensate a loss of face‐to‐face contact (Teo et al., 2015, 2019). Related to this, several COVID‐related studies indicate that time spent in front of the computer and for digital connection was even related to lower well‐being and depression during lockdown (Ellis et al., 2020; Stieger et al., 2021). On the other hand, in times of deprivation such as during a lockdown, digital or phone contact may still be better than having no social contact at all (Kushlev & Leitao, 2020, Waytz & Gray, 2018). In line with this assumption, a longitudinal study during the spring 2020 lockdown in Italy showed that online connections protected individuals from psychological distress during restrictive lockdown periods in which individuals were forced to isolate (Marinucci et al., 2022; Pancani et al., 2021). This was especially true for individuals with few face‐to‐face interactions, whereas individuals with more face‐to‐face interactions did not profit from additional online contact. It is thus possible that social contact via phone or computer might primarily be helpful in satisfying the basic needs of those who had little or no direct face‐to‐face communication during the lockdown. In contrast, individuals who managed to maintain a high frequency of face‐to‐face communication even during lockdown might be satisfied in their needs already and not depend upon additional sources of contact via phone or digital devices.

## SUMMARY RESEARCH OBJECTIVES

We conducted a longitudinal study during the onset of the pandemic in Germany to investigate *who* experienced a reduction in social contact as a consequence of social distancing measures, as well as *how* these reductions of social contact as well as shifts in the mode of communication *directly* predict need satisfaction (as a proximal consequence) and *indirectly* predict the development of well‐ and ill‐being (as a distal consequence) through need satisfaction. Our specific research objectives were as follows:

Our first research objective was to identify who would be particularly at risk of experiencing changes in social contact during the lockdown. Particularly, we investigated whether age, living conditions (alone vs. with other people) and working environment (on‐site vs. home office vs. pandemic‐related inability to work) were linked to pandemic‐related changes in the quantity of social contact and mode of communication. We also tested for interactions between these potential risk factors in an exploratory fashion.

As a second research objective, we investigated associations between the *quantity* of individuals' social contact and the development of well‐ and ill‐being. In general, we assumed that the quantity of social contact would be positively associated with satisfaction of the basic needs for autonomy, competence, and relatedness. We hypothesized that over time, need satisfaction would be positively associated with the development of life satisfaction and negatively with the development of anxiety and depression.

**FIGURE 2:**
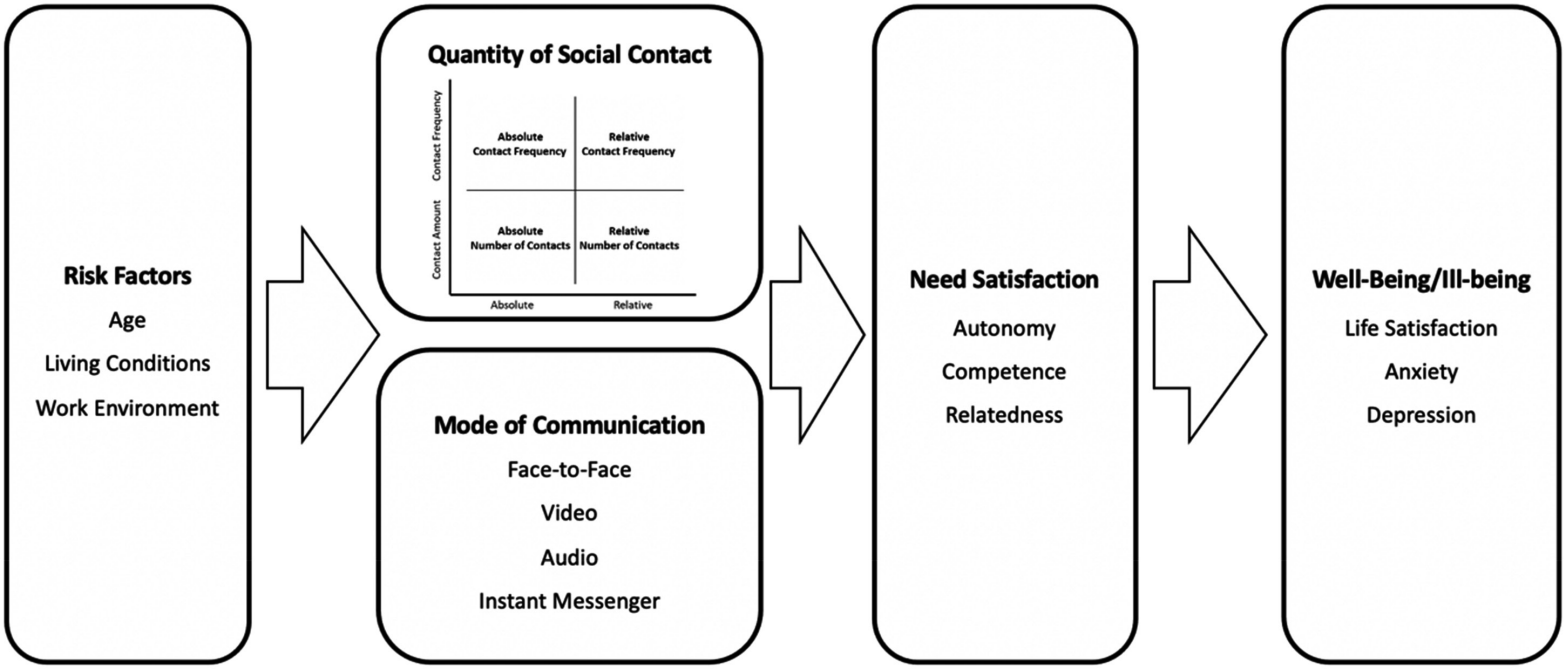
Conceptual process model of social contact

Our third research objective was to investigate whether and how the *mode of communication* was associated with need satisfaction and indirectly with the development of well‐ and ill‐being. We assumed that face‐to‐face social contact would be more strongly tied to need satisfaction than any other modes of communication (i.e. via video, phone, or instant messenger). Additionally, we investigated whether other modes of communication could compensate for a lack of face‐to‐face contact (moderation hypothesis). Our full process model of the postulated associations between different aspects of social contact, need satisfaction and well‐being is depicted in Figure 2.

Research questions and the study design were preregistered on https://aspredicted.org/c3fa4.pdf. The present contribution focusses on results and measures described in research question 2 in the pre‐registration,^1^ see Rudert and Janke (2021) for results and measures described in research question 1. Data from the study (relatedness and donation behaviour) were further used within another unrelated publication (Klein & Rudert, 2021).

## METHOD

We conducted a survey study within the German population with two time points, starting on 26 March 2020. This was 4 days after the German government had imposed nationwide contact restrictions that prohibited meetings of more than two people from different households and required people to keep a minimum distance of 1.5 metres in public. The IRB of the University of Koblenz‐Landau approved the survey (framework ‘Psychological Effects of the COVID‐19 pandemic’, IRB Nr.: 2020_255). Our complete analysis scripts, outputs, the relevant data and supplemental material (Appendix S1) for this contribution are provided at https://osf.io/2jgy3/.

### Participants

We advertised the survey as a study on psychological consequences of the COVID‐19 pandemic. To reach as many potential participants as possible, we used a snowball sampling strategy. Members of the research team promoted the survey via occupational and recreational mailing lists, as well as regional and occupational Facebook groups. Subscribers to these mailing lists and online groups were asked to distribute the study link within their own social networks. The survey was further advertised in a press release of the University of Koblenz‐Landau that was picked up by several newspapers.

The minimum age was 15 years, and participants had to reside in Germany at the time of the survey. Each study wave was online for a time span of a week, and we recruited as many participants as we could within that time span. Participants responded to the first questionnaire between March 26 and April 2 (T1) and were invited via email to participate at the second time point between April 16 and April 23 (T2). In total, 1907 individuals participated at the first time point, out of which 1342 persons also responded to the second questionnaire (dropout rate = 29.63%). Attrition analyses showed that individuals who did not participate at the second time point differed from those who participated again, *Hotelling's T* = .28, *F* (15, 1835) = 3.38, *p* < .001, η^2^ = .03. While the observed attrition effects were rather small, individuals who dropped out reported slightly lower magnitudes of autonomy (η^2^ < .01) and relatedness (η^2^ = .01), a lower absolute frequency of social contacts (η^2^ = .01) as well as less face‐to‐face communication (η^2^ = .01) and video communication (η^2^ < .01). They were also more likely to be male, χ^2^(1) = 4.69, *p* = .030.

We could match the data of both time points for 97.09% of the participants taking part at the second time point (1303 fully matched data sets). Of all participants, 76.5% were women, 22.6% were men, ten participants were gender‐diverse, and seven chose not to respond (*M*_*age*_ = 36.35, *SD* = 13.44, Range: 16–89). Most participants (82.5%) had a general qualification for university attendance or a higher education degree.

Of all participants, 53.5% reported living together with their partner, 24.4% with their own children, 16.2% with their parents, 10.9% with other family members, 5.2% with friends, 2.5% with fellow students and .3% with work colleagues. Overall, 19% of the sample were living alone. At the first time point, 39.8% of all participants reported that they had worked from home due to the pandemic, 20.1% of all participants indicated that they had worked at their workplace, 10.2% that they could not work because of the pandemic and 6.0% that they had worked from home as usual.

### Measures

All reported variables were measured at both time points. However, we solely relied on data from T1 for risk factors, facets of social contact and need satisfaction (proximal response). Only the changes in well‐being were modelled using data from both time points (distal response).

#### Social contact: Quantity of social contact

Participants answered four items assessing (1) how often they had social contact in the last 7 days (*absolute contact frequency*; 5‐point scale, 1 = *not at all*, 2 = *once per week* 3 = *several times per week*, 4 = *once per day*, 5 = *several times per day*), (2) whether this was more or less than normal (*relative contact frequency*; 7‐point scale, 1 = *much less contact*, 4 = *just as much contact* 7 = *much more contact*), (3) with how many people they had contact within the last 7 days (*absolute number of social contacts*, 5‐point scale, 1 = *no one*, 2 = *with one person*, 3 = *2–5 persons*, 4 = *6–20 persons*, 5 = *more than 20 persons*), and again (4) whether these were more or less people than normal (*relative number of social contacts*; 7‐point scale, 1 = *contact with far fewer people*, 4 = *contact with just as many people*, 7 = *contact with far more people*).

#### Social contact: Mode of communication

We asked participants how often they had engaged in (1) direct, face‐to‐face communication, (2) video communication, (3) phone communication and (4) instant messenger communication within the last 7 days. Participants answered these four questions using the same 5‐point scale that had been used to measure absolute contact frequency (1 = *not at all*, 2 = *once per week*, 3 = *several times per week*, 4 = *once per day*, 5 = *several times per day*).

#### Need satisfaction

Relatedness, autonomy, and competence were assessed with the Balanced Measure of Psychological Needs with six items per need (Sheldon & Schüler, 2011; 7‐point scale, 1 = *not at all*; 7 = *very much*). Three items per scale were inverted and reverse‐coded prior to aggregation. Example items were ‘I felt close and connected with other people who are important to me’ (relatedness; α = .70), ‘I struggled doing something I should be good at’ (reverse‐coded; competence; α = .70), and ‘I was free to do things my own way’ (autonomy; α = .70).

#### Life satisfaction

Life Satisfaction within the previous 7 days was assessed with a single item, asking ‘Overall, how satisfied are you with your life right now’ (Beierlein et al., 2015; 10‐point scale, 1 = *not satisfied at all*, 10 = *completely satisfied*).

#### Anxiety

Anxiety was assessed with 12 items measuring tendencies to worry within the previous 7 days, for example ‘Once I started worrying, I could not stop’ (Barenbrügge et al., 2014; 5‐point scale, 1 = *not at all*, 5 = *very much*, α_TP1_ = .95; α_TP2_ = .95).

#### Depression

Depression was assessed with eight items measuring subclinical depression, asking participants how often several statements had applied to them within the previous 7 days, for example ‘I looked to the future without hope’ (Mohr & Müller, 2014; 7‐point scale, 1 = *never*, 7 = *nearly always*; α_TP1_ = .89; α_TP2_ = .90).

### Analytic strategy

We investigated our research objectives in three subsequent steps: First, we analysed potential antecedents for a loss of social contact. We conducted 1F‐MANOVAS with living condition (living alone vs. with other people) and work environment (on‐site vs. home office vs. inability to work due to the pandemic) as independent variables and all eight measures for the quantity of social contact and mode of communication as dependent variables. Furthermore, we investigated correlations of all social contact indicators with age. As exploratory analyses, we also investigated interactions between the three postulated risk factors.

Second, we conducted path analyses to investigate whether indicators of social contact quantity (i.e. relative and absolute contact frequency, relative and absolute number of contacts) (a) predicted need satisfaction at T1 and (b) indirectly predicted changes in life satisfaction, anxiety, and depression over time via need satisfaction. To model actual change in life satisfaction, anxiety, and depression over time, we controlled for these variables at T1. We allowed for associations between the three psychological needs and between the different indicators of social contact. To reduce model complexity, we conducted three separate models for life satisfaction, anxiety and depression.

Third, we repeated the described analysis for different modes of social communication. We also investigated whether alternative modes of communication could compensate for low face‐to‐face contact by testing whether any alternative mode of communication would moderate the strength of association between the frequency of face‐to‐face contact and need satisfaction.

All path analyses were conducted with Mplus Version 8.6 (Muthén & Muthén, 1998–2017). We applied the full information maximum likelihood (FIML) to handle missing data when estimating the model parameters. We used the robust maximum likelihood estimator (MLR), which allows robust estimations of model parameters even when deviations in multivariate normality occur. To estimate model fit, we use a combination of misfit (SRMR, RMSEA) and fit indices (CFI, TLI; see Hu & Bentler, 1999).

## RESULTS

**FIGURE 3:**
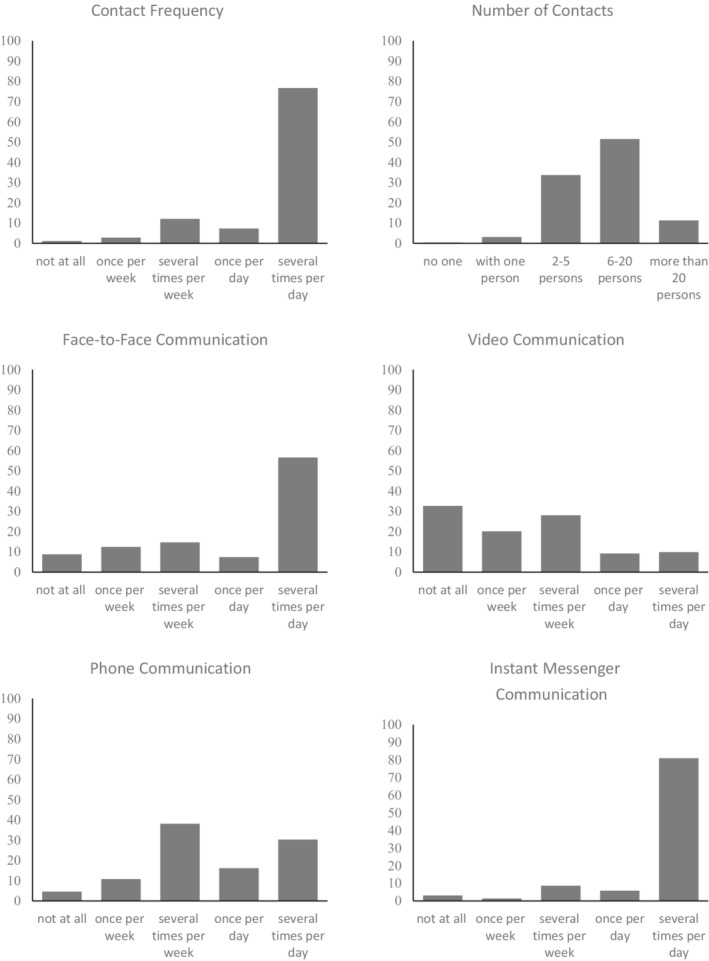
Descriptive relative frequencies (in percentage) of social contact quantity and communication mode

Relative frequencies for the different measures of social contact are depicted in Figure 3. In general, most of our participants still had contact with other people on a daily basis (84%) and had interacted with more than two other persons within the last 7 days (96.5%). However, the majority also reported a relative reduction in contact frequency (60.9% compared to 16.3% experiencing no change and 22.8% experiencing an increase in social contact) and a relative reduction in the number of contacts (60.3% compared to 22.9% experiencing no change and 16.9% experiencing an increase). As for the mode of communication, 64.0% reported having engaged in face‐to‐face communication on a daily basis. Additionally, 86.8% reported engaging in instant messenger communication, 46.5% in phone communication and 19.1% in video communication on a daily basis. Zero‐order associations between all variables are depicted in a supplementary table (Appendix S1) under https://osf.io/2jgy3/.

When looking at the indicators of well‐being and ill‐being between the two time points, we found that on average, life satisfaction had slightly increased, *t*(1301) = −2.30, *p* = .022, *d* = .06, whereas the mean score of reported anxiety had decreased, *t*(1301) = 6.90, *p* < .001, *d* = −.13 between the first and the second time point. On average, there was no change in depression, *t*(1301) = .18, *p* = .854.

### Step 01: Antecedents of contact quantity and mode of communication

#### Age

Age was especially associated with the chosen device for non‐face‐to‐face communication. Older participants were less likely to use video communication (*r* = −.19, *p* < .001) or instant messenger communication (*r* = −.23, *p* < .001), but were more likely to rely on phone communication (*r* = .26, *p* < .001). There was no association between age and the frequency of face‐to‐face contact. Regarding the quantity measures, we found a small association indicating a stronger decrease in the relative number of contacts for older compared with younger participants (*r* = −.05, *p* = .025), but no significant associations with the absolute number of contacts or contact frequency, smallest *p* = .055.

#### Living conditions

Results of a 1‐F MANOVA showed an effect of living with other persons versus living alone on quantity of social contact and mode of communication during lockdown, *Hotelling's T* = .20, *F* (8, 1839) = 44.85, *p* < .001, η^2^ = .16 (for descriptive and ANOVA statistics, see Table 1). This was mainly driven by a mode indicator, namely that participants living alone (vs. not) reported a lower frequency of face‐to‐face communication. Participants living alone (vs. not) instead reported a slightly higher frequency of communication via phone; but not via video or instant messenger. As for the quantity indicators, participants living with others reported a higher absolute frequency of social contact than individuals living alone. However, individuals living with others also reported a stronger decrease in relative contact frequency during the pandemic than individuals living alone. Neither the absolute nor the relative number of contacts differed significantly due to living conditions. We found no significant interaction effects between age and living conditions, *Hotelling's T* = .01, *F* (8, 1820) = 1.27, *p* = .256.

**TABLE 1:** Descriptive and inferential statistics for social contact by living conditions

Social contact dimension	Living conditions		*F* (1, 1846)	*p*	η^2^
	Alone	With others			
Absolute contact frequency	4.32 (.99)^a^	4.62 (.85)^b^	34.37	<.001	.018
Absolute number of contacts	3.67 (.72)^a^	3.72 (.72)^a^	1.37	.242	.001
Relative contact frequency	3.46 (1.76)^a^	3.10 (1.75)^b^	12.32	<.001	.007
Relative number of contacts	3.13 (1.65)^a^	2.95 (1.64)^a^	3.47	.062	.002
Face‐to‐face communication	2.77 (1.24)^a^	4.18 (1.32)^b^	333.77	<.001	.153
Video communication	2.38 (1.29)^a^	2.45 (1.30)^a^	.93	.335	.001
Phone communication	3.71 (1.17)^a^	3.54 (1.15)^b^	6.21	.013	.003
Instant messenger communication	4.62 (.92)^a^	4.60 (.93)^b^	.06	.807	.000

*Note:* Means (standard deviations) on the social contact variables as a function of living conditions and significance tests. The superscript letters a–b represent significant differences between groups; all values in the same row that share the same letter do not differ significantly from each other; values with different letters do.

#### Work environment

Results of a 1‐F MANOVA showed effects of working conditions, *Hotelling's T* = .73, *F*(16, 2824) = 6.40, *p* < .001, η^2^ = .04 (for descriptive and ANOVA statistics, see Table 2). Particularly, we found significant differences in contact frequency and number of social contacts. Individuals who could not work due to the pandemic reported a lower contact frequency compared with individuals working from home, and a lower number of contacts compared with both individuals working from home and on‐site. However, there were no significant differences between working conditions regarding relative changes of social contact. Looking at the mode of communication, we found differences in the frequency of video and phone communication, with individuals working from home engaging more in both modes of communication compared with individuals who were working on‐site or could not work. We found no significant differences between working conditions regarding the frequency of instant messenger or face‐to‐face communication, nor any significant interaction effects between age and working conditions, *Hotelling's T* = .01, *F* < 1.

**TABLE 2:** Descriptive and inferential statistics for social contact by work environment

Social contact dimension	Working environment			*F* (2, 1420)	*p*	η^2^
	On‐site	From home	Unable to work			
Absolute contact frequency	4.54 (.88)^ab^	4.63 (.84)^a^	4.43 (1.03)^b^	4.41	.012	.006
Absolute number of contacts	3.76 (.75)^a^	3.78 (.72)^a^	3.55 (.68)^b^	7.42	.001	.010
Relative contact frequency	3.03 (1.70)^a^	3.18 (1.75)^a^	3.09 (1.92)^a^	.96	.384	.001
Relative number of contacts	2.86 (1.60)^a^	2.99 (1.61)^a^	2.82 (1.72)^a^	1.29	.276	.002
Face‐to‐face communication	3.95 (1.36)^a^	3.97 (1.42)^a^	3.82 (1.40)^a^	.85	.427	.001
Video communication	2.07 (1.27)^a^	2.73 (1.29)^b^	2.31 (1.22)^c^	37.28	< .001	.050
Phone communication	3.55 (1.18)^a^	3.74 (1.09)^b^	3.39 (1.16)^b^	9.42	< .001	.013
Instant messenger communication	4.65 (.84)^a^	4.64 (.91)^a^	4.63 (.85)^a^	.05	.948	.000

*Note*. Means (standard deviations) on the social contact variables as a function of work environment and significance tests. The letters a–b represent significant differences (*p* < .05) between groups; all values in the same row that share the same letter do not differ significantly from each other; values with different letters do.

#### Exploratory analysis: Work environment × living conditions

Interestingly, when investigating work environment and living conditions within a 2F MANOVA, we found a significant interaction effect, *Hotelling's T* = .43, *F* (16, 2798) = 3.77, *p* < .001, η^2^ = .02 (see Table 3 for all descriptive and ANOVA statistics). The interaction was driven by absolute contact frequency and face‐to‐face communication. Simple main effect analyses revealed that in the group of individuals living alone, those who could not work reported a lower contact frequency compared to those working from home or on‐site. In contrast, among individuals living with others, there were no significant associations between work environment and contact frequency.

**TABLE 3:** Descriptive and inferential statistics for social contact by living conditions × work environment

Social contact dimension	Living conditions	Work environment			*F* (5, 1407)	*p*	η^2^
		On‐site	From home	Unable to work			
Absolute contact frequency	With others Alone	4.60 (.84)^a^ 4.34 (.97)^b^	4.67 (.82)^a^ 4.54 (.90)^b^	4.43 (.97)^a^ 3.83 (1.17)^c^	8.04	<.001	.028
Absolute number of contacts	With others Alone	3.80 (.77)^a^ 3.65 (.69)^a^	3.77 (.72)^a^ 3.80 (.72)^a^	3.59 (.64)^b^ 3.34 (.86)^b^	4.18	.001	.015
Relative contact frequency	With others Alone	2.92 (1.74)^a^ 3.42 (1.55)^b^	3.15 (1.75)^a^ 3.31 (1.76)^ab^	3.01 (1.90)^a^ 3.55 (2.01)^ab^	2.11	.062	.007
Relative number of contacts	with others alone	2.13 (1.60)^a^ 3.10 (1.60)^a^	2.97 (1.60)^a^ 3.06 (1.64) ^a^	2.79 (1.76)^a^ 3.00 (1.49) ^a^	1.78	.318	.004
Face‐to‐face communication	With others Alone	4.11 (1.34)^a^ 3.40 (1.30)^c^	4.31 (1.25)^b^ 2.56 (1.21)^d^	4.06 (1.35)^a^ 2.52 (.91)^d^	61.98	<.001	.180
Video communication	With others Alone	2.10 (1.28)^a^ 2.02 (1.23)^a^	2.73 (1.29)^b^ 2.72 (1.30)^b^	2.32 (1.23)^a^ 2.24 (1.15)^a^	14.28	<.001	.048
Phone communication	With others Alone	3.54 (1.19)^a^ 3.58 (1.17)^a^	3.72 (1.09)^b^ 3.88 (1.11)^b^	3.33 (1.16)^a^ 3.76 (1.12)^a^	5.13	<.001	.018
Instant messenger communication	With others Alone	4.66 (.84)^a^ 4.65 (.86)^a^	4.64 (.91)^a^ 4.68 (.88)^a^	4.63 (.87)^a^ 4.62 (.73)^a^	.12	.989	.000

*Note:* Means (and standard deviations) on the social contact variables as a function of living conditions and work environment and significance tests of the interaction. The superscript letters a–d represent significant differences (*p* < .05) between groups; all values in the same row or column that share the same letter do not differ significantly from each other; values with different letters do.

Regarding the mode of communication, among individuals living with others, those working from home even reported *more* face‐to‐face communication than those working on‐site or those who could not work. In contrast, in the group of individuals living alone, those working from home or could not work reported *less* face‐to‐face contact than those working on‐site.

### Step 02: Quantity of social contact and indicators of well‐ and ill‐being

**FIGURE 4:**
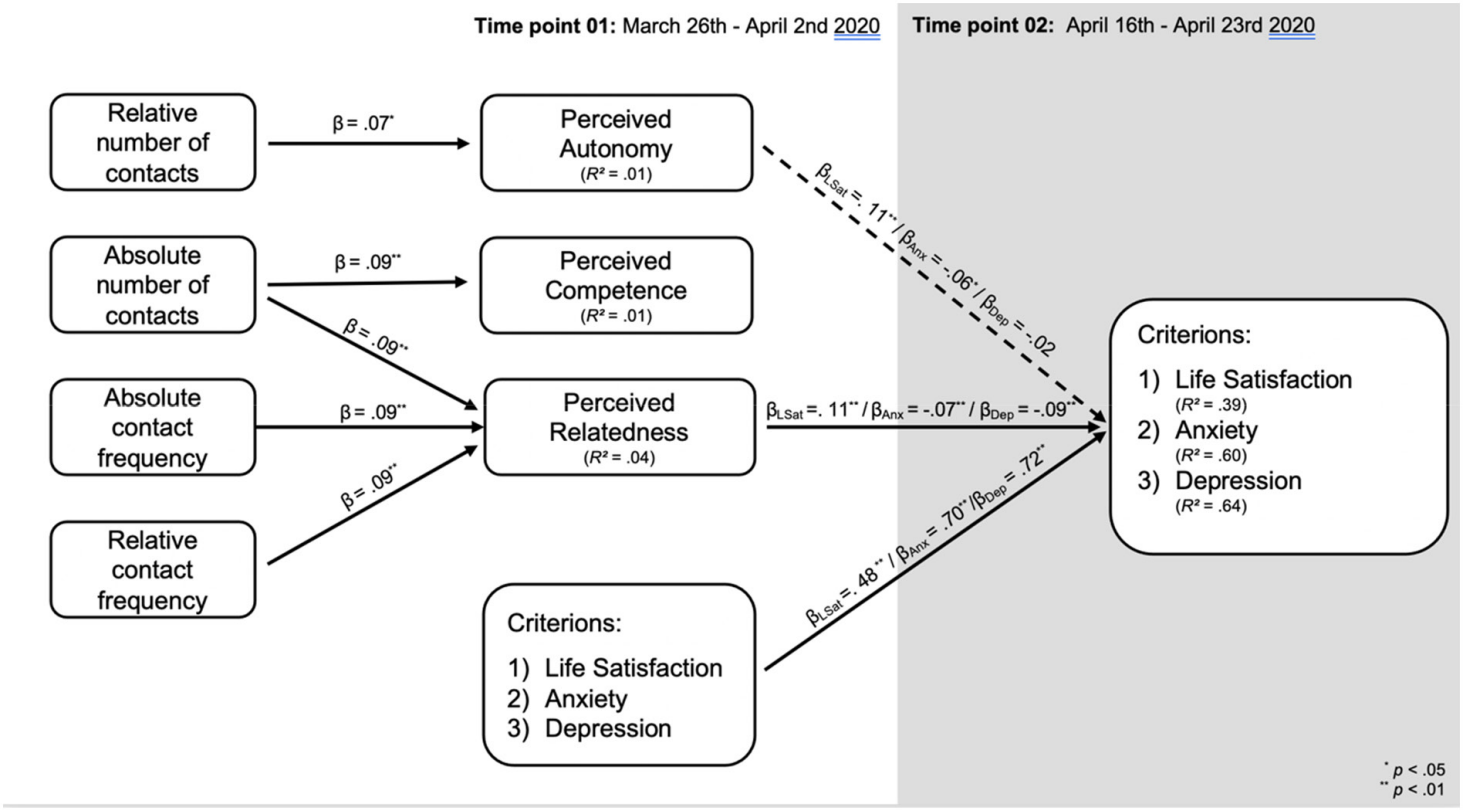
Structural model for quantity of social contact. β_Anx_, Beta‐coefficients derived from the path model with anxiety as a criterion; β_Dep_, Beta‐coefficients derived from the path model with depression as a criterion; and β_LSat_, Beta‐coefficients derived from the path model with life satisfaction as a criterion. The depicted model only includes direct paths that were statistically significant at *p* < .05 for better comprehensibility and reflects an integration of all three path models. All undirected associations and non‐significant path can be directly derived from the Appendix S1

According to the criteria by Schermelleh‐Engel et al. (2003), all three path models showed a very good model fit with SRMR < .01, RMSEA < .02, CFI > .99, TLI > .99. All direct paths that reached statistical significance are depicted in Figure 4. For better comprehensibility, we report associations between measures of social contact and cross‐sectional associations between need satisfaction and the criteria at T1 in the supplemental material (Appendix S1) under https://osf.io/2jgy3/.

Regarding the direct paths, we found that the absolute number of social contacts, absolute contact frequency and relative change in contact frequency predicted experienced relatedness. Relative change in the number of social contacts did not predict relatedness, but perceived autonomy. The absolute number of social contacts was also positively associated with perceived competence.

Experienced relatedness at T1 predicted an increase in life satisfaction between T1 and T2 and a decrease in anxiety and depression. Experienced autonomy at T1 predicted an increase in life satisfaction and a decrease in anxiety. Experienced competence at T1 was not associated with changes in well‐being. Looking at indirect effects, indicators of the quantity of social contact generally predicted measures of well‐being and ill‐being through experienced relatedness, but not via experienced autonomy or competence. All statistically significant indirect effects are depicted in Table 4.

**TABLE 4:** Indirect effects of social contact (T1) via need satisfaction (T1) on change in well‐being (T2)

Criterion = Life Satisfaction	
Quantity: Absolute number of contacts ➔ Relatedness ➔ Life Satisfaction	β_indirect_ = .01, *p* = .015
Quantity: Absolute frequency of contact ➔ Relatedness ➔ Life Satisfaction	β_indirect_ = .01, *p* = .013
Quantity: Relative frequency of contact ➔ Relatedness ➔ Life Satisfaction	β_indirect_ = .01, *p* = .023
Mode: Face‐to‐face communication ➔ Relatedness ➔ Life Satisfaction	β_indirect_ = .02, *p* = .004
Mode: Phone communication ➔ Relatedness ➔ Life Satisfaction	β_indirect_ = .01, *p* = .004

Criterion = Anxiety	
Quantity: Absolute number of contacts ➔ Relatedness ➔ Anxiety	β_indirect_ = −.01, *p* = .031
Quantity: Absolute frequency of contact ➔ Relatedness ➔ Anxiety	β_indirect_ = −.01, *p* = .028
Quantity: Relative frequency of contact ➔ Relatedness ➔ Anxiety	β_indirect_ = −.01, *p* = .043
Mode: Face‐to‐face communication ➔ Relatedness ➔ Anxiety	β_indirect_ = −.01, *p* = .013
Mode: Phone communication ➔ Relatedness ➔ Anxiety	β_indirect_ = −.01, *p* = .016

Criterion = Depression	
Quantity: Absolute number of contacts ➔ Relatedness ➔ Depression	β_indirect_ = −.01, *p* = .009
Quantity: Absolute frequency of contact ➔ Relatedness ➔ Depression	β_indirect_ = −.01, *p* = .007
Quantity: Relative frequency of contact ➔ Relatedness ➔ Depression	β_indirect_ = −.01, *p* = .019
Mode: Face‐to‐face communication ➔ Relatedness ➔ Depression	β_indirect_ = −.01, *p* = .001
Mode: Phone communication ➔ Relatedness ➔ Depression	β_indirect_ = −.01, *p* = .002

*Note:* This table only includes statistically significant at *p* < .05 indirect effects that can be derived from the path analyses.

### Step 03: Modes of communication and indicators of well‐ and ill‐being

**FIGURE 5:**
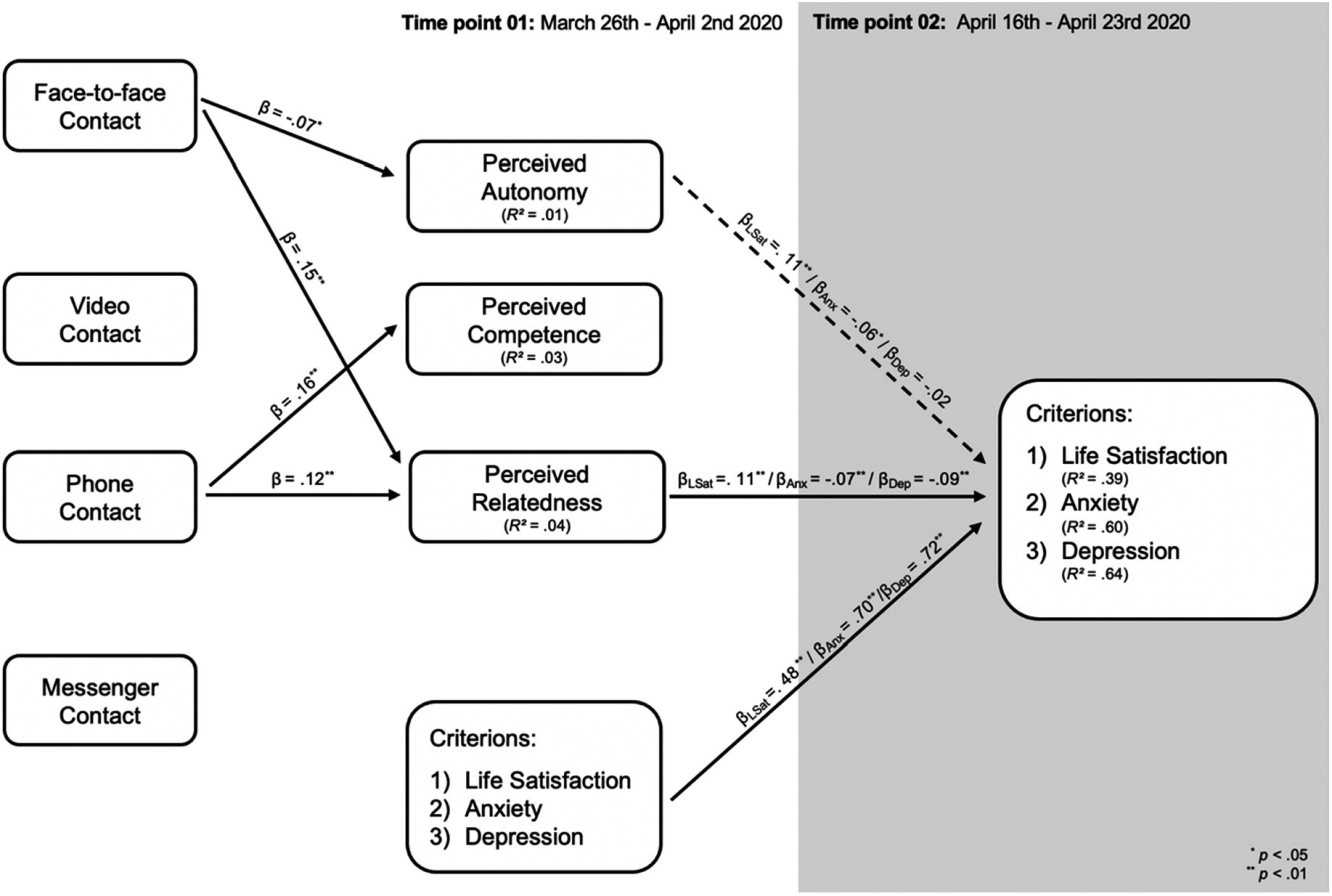
Structural model for mode of communication. β_Anx_, Beta‐coefficients derived from the path model with anxiety as a criterion; β_Dep_, Beta‐coefficients derived from the path model with depression as a criterion; and β_LSat_, Beta‐coefficients derived from the path model with life satisfaction as a criterion. The depicted model only includes direct paths that were statistically significant at *p* < .05 for better comprehensibility and reflects an integration of all three path models. Associations between modes of communication as well as non‐significant paths can be directly derived from the Appendix S1

The path analyses on the importance of different modes of communication showed a good model fit (see Schermelleh‐Engel et al., 2003) with SRMR < .02, RMSEA < .04, CFI > .99, TLI > .95. Statistically significant direct paths are depicted in Figure 5, and associations between modes of communication are depicted in the Appendix S1. The frequency of face‐to‐face communication as well as communication via phone predicted feelings of relatedness and indirectly change in all three indicators of well/ill‐being between T1 and T2 via relatedness (see Table 4). Furthermore, communication via phone positively predicted perceived competence, whereas face‐to‐face communication negatively predicted perceived autonomy. However, none of these direct associations translated into statistically significant indirect effects on indicators of well/ill‐being. Furthermore, the degree of explained variance was not significant for experienced autonomy, *R* ^*2*^ = .006, *p* = .106. We found no interaction between face‐to‐face communication and other modes of communication, indicating that alternative ways of communication could not compensate for a lack of face‐to‐face communication.

## GENERAL DISCUSSION

One of the most prominent societal changes in the wake of the COVID‐19 pandemic has been the mandatory reduction in social contact to contain the spread of the virus. In a longitudinal study, we investigated social contact at the onset of the COVID‐19 pandemic in Germany, focussing on (1) the identification of risk factors for declined social contact as well as associations between (2) reductions in social contact and (3) different modes of communication with need satisfaction, well‐being and ill‐being over time. Individuals who lived alone and were not able to work due to the pandemic were particularly at risk to experience a lower frequency of social contact and less face‐to‐face communication during the onset of the pandemic. A lower quantity of social contact was subsequently associated with decreased life satisfaction, higher anxiety and depression scores a few weeks later mediated via experienced relatedness at the first time point. A higher frequency of face‐to‐face communication and phone communication was positively associated with relatedness and changes in well‐being, while digital communication (video communication and instant messenger) was not.

### Risk factors for impaired social contact

Most participants reported a reduction in the frequency and number of social contacts during lockdown. However, simple generalizations do not seem to be appropriate, given that about 17%–23% of the sample even reported having *more* social contact than usual. Thus, the data point to the necessity to distinguish between certain groups when investigating social contact and isolation during the lockdown.

It is not surprising that especially individuals living alone reported a lower degree of face‐to‐face communication as well as a lower frequency of social contact. However, it is interesting to note that individuals living with others reported a stronger relative *decline* in contact frequency compared with individuals living alone. One explanation is that individuals who were used to being alone before the pandemic were less affected by pandemic‐related changes (see also Heidinger & Richter, 2020; Lee et al., 2020).

Compared with individuals' living conditions, the work environment seemed to be less strongly linked to reported social contact. However, individuals who could not work due to the pandemic *and* were also living alone reported the lowest contact frequency of all investigated groups. Potentially, this group might be at a particular risk for social isolation in times when private gatherings with others are discouraged or prohibited.

While living and working status proved to be important risk factors in our sample, age seemed to be mostly associated with a preference for certain modes of communication, but not with contact reduction. However, note that our sample was relatively young on average (*M*_age_ = 36.35), with few individuals belonging to older age groups 60+. Studies with larger samples of adults >60 years indeed showed stronger increases in loneliness for older compared with younger individuals (e.g. Buecker et al., 2020; Luchetti et al., 2020).

### Psychological mechanisms linking social contact and well‐being

Interestingly, we found no overall decrease in well‐being during the lockdown. In fact, life satisfaction increased and anxiety decreased between the first and the second measurement point, while depression levels did not change on average. While this might appear surprising, note that our study did not include a pre‐pandemic measure and thus, the overall improvement might indicate that after an initial shock during the first days of lockdown, most individuals managed to cope and get used to the ‘new normal’ (Prati & Mancini, 2021). However, this was clearly not the case for all individuals: Our data suggest that particularly the ability to uphold social contacts during the imposed lockdown was crucial for the development of well‐being. This was due to the positive association of social contact with experienced relatedness, which was in turn associated with the trajectory of changes in life satisfaction, anxiety and depression several weeks later. Autonomy and competence, however, were only weakly linked to social contact, and we also observed no significant indirect effects linking social contact indicators with well‐being via autonomy or competence. Rather than indicating an overall negative relation between reduced social contact and need satisfaction, the results thus suggest the necessity to differentiate between needs; with relatedness being focal for explaining associations between indicators of social contact and well‐being.

Overall, our findings contribute to contemporary debates on well‐being during the pandemic: While empirical studies suggest that the well‐being of the general population is at least somewhat resilient to temporary lockdown measures (e.g. Cantarero et al., 2021; Prati & Mancini, 2021; van Tilburg et al., 2021), our findings contribute to a deeper understanding on whether and particularly how (maintaining) social contact in such times can act as protective factor that enhances this resilience.

### Digital and face‐to‐face‐communication during lockdown

Similar to associations between the quantity of social contact and well‐being, associations between the mode of communication and well‐being were only mediated via relatedness, but not autonomy and competence, once again stressing the importance of this particular need during the lockdown period. While more face‐to‐face and phone communication were associated with higher relatedness, and, indirectly, with increased well‐being, the frequency of video communication or instant messenger communication was not. The finding that phone but not video communication was associated with relatedness might appear puzzling at first. This is particularly true given the absence of significant differences between working and non‐working individuals, which makes it unlikely that our findings merely reflect the absence of a positive effect of work‐related video calls. However, phone calls are usually conversations between two persons that might provide high intimacy and quality of communication and, thus, foster relatedness. In contrast, video communication and text messaging are often used for group conversations, in which individuals may be less able to engage with others in line with their inner needs. Future research may, thus, want to further inquire which conditions facilitate the observed positive associations for communication via phone. In addition, it might be interesting to investigate whether increasing familiarity with certain communication devices plays a role for the observed associations. Many individuals were less familiar with video communication at the beginning of the pandemic compared with one or two years after; thus, respective associations might be prone to change over time.

Despite the positive association between relatedness and phone communication, we found no evidence that any kind of non‐face‐to‐face communication could compensate for a lack of face‐to‐face communication. This differs from findings by Marinucci et al. (2022) who found a respective interaction during the strictest phase of the Italian lockdown. A potential explanation for these diverging findings might be that the German lockdown was less strict than the Italian lockdown (e.g. Germans were still allowed to leave the house and meet with one person outside of their own household). The usefulness of (digital) compensation alternatives might thus be limited to cases of severe isolation.

### Methodological and practical considerations

Within the present contribution, we focussed on the quantity of social contact and mode of communication, as we assumed those two facets to be most likely affected by the lockdown. An additional facet of social contact that future research might wish to incorporate is the *quality* of one's social connections, meaning the closeness of the people one had contact with (Fiorillo & Sabatini, 2011). Quality of social connections could moderate the association between the quantity of social contact and relatedness in the sense that social contact might be primarily associated with increased relatedness when interacting with close others.

We further note that our study was not based on a representative sampling strategy, with young individuals and women being overrepresented in the sample. Due to the online‐based sampling strategy, the sample does not contain non‐Internet users, which might have particularly contributed to the age bias. Overall, this sampling bias might have affected the descriptive findings, but could also have impacted observed relations (e.g. the limited associations of social contact measures with age might be due to the underrepresentation of older individuals within our sample). Albeit attrition effects were generally small, we found that individuals who engaged in less social communication were more likely to drop out between the two time points. The parameter estimations might thus underrepresent individuals that experienced the least amount of social contact.

An important strength of our research is the longitudinal design. Research investigating well‐being and psychological needs during the COVID‐19 pandemic with cross‐sectional data does not allow for an investigation of temporal associations as well as change over time. In contrast, our research directly addresses temporal order between potential predictor variables and well‐being as a criterion. As an important limitation, our data do not allow us to draw causal inferences, as temporal order is a pre‐condition, but not a proof of causation. Particularly, unobserved third variables may affect both social contact as well as relatedness and our well‐being indicators. Furthermore, our study was limited to two time points. This means that we cannot investigate associations of predictor variables with change in mediator variables and associations of the mediator with change in the criteria simultaneously. However, from a theoretical standpoint, an additional time lag would likely not have led to additional insights on the association between social contact and need satisfaction. The assumed decline in experienced relatedness following contact restrictions is most likely an immediate response, which means that it is particularly difficult to observe temporal order using survey designs that investigate longer time periods. In contrast, experimental studies that allow to observe immediate reactions seem more sensible for this purpose and in fact show that contact deprivation, such as ostracism, decreases relatedness (see Legate et al., 2013).

Our study highlights the importance of maintaining social contacts during states of emergency (e.g. lockdowns) and paying specific attention to certain risk groups. Particularly, individuals who live alone and cannot work may suffer from low contact during lockdowns. Critically, with social contact, the importance of psychological health clashes with the importance of physical health during a pandemic: While individuals' mental health and well‐being may improve during face‐to‐face interactions with others, these interactions put them at a higher risk of becoming infected. As an additional complication, our study indicates that often‐praised digital alternatives might not provide sufficient compensation for a loss of face‐to‐face communication.

An important question with strong practical implications is how the investigated processes might unfold in even longer time spans. Our data were assessed during the first lockdown in Germany, which ended after approximately 2 months in May but was followed by a significantly longer lockdown starting in October 2020 and continuing until spring/summer 2021. During the first lockdown, individuals might have still been motivated to maintain or even increase social contact via communication alternatives. However, during a longer lockdown, people might have gotten used to the ‘new normal,’ and grown weary of constant phone and video calls. For individuals with strong social ties who live with others, this might have resulted in focussing increasingly on their close social circle. In contrast, individuals with weaker social ties might easily be forgotten, and the limited possibilities within the lockdown might make it hard to make new friends. In a worst‐case scenario, these new social structures might solidify and continue even after the lockdown, with some individuals having become too isolated and depressed to reach out and others having long moved on. Politicians and decision‐makers should pay attention to these potential detrimental effects and offer social events and opportunities for face‐to‐face (re)connection once the pandemic is over.

## CONCLUSION

Living alone and not being able to work during the pandemic turned out to be important risk factors for a lower quantity of social contact during the onset of the COVID‐19 pandemic in Germany. Less social contact further predicted decreased life satisfaction, increased anxiety and depression several weeks later via decreased relatedness. While face‐to‐face and phone communication positively predicted relatedness and well‐being, digital communication alternatives did not. These findings do not only have implications for social life during lockdowns, but also for the restoration of a society with functioning social ties after the end of pandemic‐related regulations. It is important to motivate individuals to restore social relationships with those that are most likely to have been forgotten due to their living or working conditions. Particularly, writing a well‐intended text message to individuals craving genuine face‐to‐face communication might not be sufficient.

## AUTHOR CONTRIBUTIONS

**Selma C. Rudert:** Conceptualization; Data curation; Formal analysis; Investigation; Methodology; Project administration; Resources; Visualization; Writing – original draft; Writing – review & editing. **Stefan Janke:** Conceptualization; Formal analysis; Investigation; Methodology; Visualization; Writing – review & editing.

## CONFLICT OF INTEREST

All authors declare no conflict of interest.

### OPEN RESEARCH BADGES

This article has earned an Open Data badge for making publicly available the digitally‐shareable data necessary to reproduce the reported results. The data is available at [https://osf.io/2jgy3/].

This article has earned a Preregistered Research Designs badge for having a preregistered research design, available at [https://aspredicted.org/c3fa4.pdf].

## Supporting information


 

Appendix S1

## Data Availability

The data that support the findings of this study are openly available at https://osf.io/2jgy3/.

## Footnotes

^1^
In the preregistration, we stated that we would also investigate associations between social contact with mood and loneliness. Upon data analysis, we realized that investigations into mood would have called for a more fine‐grained research design with more time points because shifts in mood likely represent a very immediate reaction to decreased need satisfaction (Ryan et al., 2010). As for loneliness, data analysis showed a strong construct overlap between loneliness and decreased relatedness (*r* = −.62, *p* < .001). Thus, we decided to focus solely on relatedness within the manuscript. We offer additional analyses in the supplemental material, in which we replaced relatedness with loneliness (the result pattern was mostly identical). We also had considered extraversion and competencies with communication devices as potential moderator variables. As neither of these variables moderated any associations, we refrain from discussing them within the manuscript for reasons of simplicity (analysis scripts and outputs can be found in the supplemental material).
