# Supplementary material for: Call me maybe: Risk factors of impaired social contact during the COVID‐19 pandemic and associations with well‐being
Source: Br J Soc Psychol. 2022 May 26:10.1111/bjso.12546. Online ahead of print. doi: 10.1111/bjso.12546 (PMC9348265; doi:10.1111/bjso.12546)
Supplement: Supplementary file 1 — Appendix S1 [file BJSO-9999-0-s001.zip › Supplemental tables and figures/Supplemental table 2 - Undirected associations for quantity.pdf]

Supplemental Table 2

Associations between measures of quantity of social contact, need satisfaction and well-being at T1.

	(1)	(2)	(3)	(4)	(5)	(6)	(7)
(1) Absolute amount of social contact							
(2) Relative amount of social contact	.36**						
(3) Absolute frequency of social contact	.29**	.16**					
(4) Relative frequency of social contact	.27**	.70**	.29**				
(5) Perceived autonomy							
(6) Perceived competence					.50**		
(7) Perceived relatedness					.49**	.47**	
(8) Anxiety	-.15*	-.04	-.05*	-.04	-.49**	-.44**	-.41**
(9) Depression	-.16**	-.06*	-.05*	-.08**	-.52**	-.61**	-.55**
(10) Life Satisfaction	.15**	.10**	.06*	.10**	.45**	.42**	.45**

Note. The table only includes associations reflecting undirected paths included in the structural models of the conducted path analyses. Associations reflecting directed paths (i.e., quantity of social contact → need satisfaction) are depicted in Figure 4. Associations with well-being indicators are exclusive to the respective path model.

** $p < .01$

* $p < .05$
