# Supplementary material for: Call me maybe: Risk factors of impaired social contact during the COVID‐19 pandemic and associations with well‐being
Source: Br J Soc Psychol. 2022 May 26:10.1111/bjso.12546. Online ahead of print. doi: 10.1111/bjso.12546 (PMC9348265; doi:10.1111/bjso.12546)
Supplement: Supplementary file 1 — Appendix S1 [file BJSO-9999-0-s001.zip › Supplemental tables and figures/Supplemental table 3 - Undirected associations for mode.pdf]

Supplemental table 3.

Associations between mode of communication and well-being at T1.

	(1)	(2)	(3)	(4)
(1) Face-to-face communication				
(2) Video communication	-.01			
(3) Communication via phone	-.01	.24**		
(4) Communication via text messenger	.06*	.19**	.16**	
(5) Anxiety	-.05*	-.01	-.02	.05*
(6) Depression	-.05**	-.01	-.11**	.07**
(7) Life Satisfaction	.08**	.08**	.07*	.01

Note. This table only includes associations reflecting undirected paths for modes of communication that were included in the structural models of the conducted path analyses. Directed paths (i.e., from face-to-face communication on need satisfaction) are depicted in Figure 5. Associations of need satisfaction with well- and ill-being at T1 are identical to the associations reported in Table 4. Associations with the three criteria are exclusive to the respective path model.

** $p < .01$

* $p < .05$
