# Supplementary material for: Call me maybe: Risk factors of impaired social contact during the COVID‐19 pandemic and associations with well‐being
Source: Br J Soc Psychol. 2022 May 26:10.1111/bjso.12546. Online ahead of print. doi: 10.1111/bjso.12546 (PMC9348265; doi:10.1111/bjso.12546)
Supplement: Supplementary file 1 — Appendix S1 [file BJSO-9999-0-s001.zip › Supplemental tables and figures/Supplemental table 1 - Zero-order correlations.pdf]

Supplemental Table 1

Descriptive statistics and correlations between quality and mode of social contact, need satisfaction and well-being

Scale	*M*	*SD*	Scale Range	(1)	(2)	(3)	(4)	(5)	(6)	(7)	(8)	(9)	(10)	(11)	(12)	(13)	(14)	(15)	(16)
(1) Absolute Contact Frequency	4.56	0.89	1–5																
(2) Absolute Contact Amount	3.70	0.72	1–5	.29**															
(3) Relative Contact Frequency	3.17	1.76	1–7	.22**	.27**														
(4) Relative Contact Amount	2.98	1.64	1–7	.16**	.36**	-.70**													
(5) Frequency Face-to-face Contact	3.90	1.41	1–5	.38**	.18**	-.04	-.01												
(6) Frequency Video Contact	2.43	1.30	1–5	.11**	.22**	.11**	.14**	-.01											
(7) Frequency Phone Contact	3.57	1.16	1–5	.22**	.22**	.17**	.13**	-.01	.24**										
(8) Frequency Text Messaging	4.60	0.93	1–5	.20**	.18**	.05	.08**	.06*	.19**	.16**									
(9) Relatedness	5.43	1.05	1–7	.14**	.14**	.13**	.11**	.15**	-.01	.10**	-.02								
(10) Autonomy	4.77	1.13	1–7	.00	.04	.08**	.10**	-.05*	-.01	.03	-.01	.49**							
(11) Competence	4.82	1.10	1–7	.04	.10**	.07**	.05*	.01	.03	.15**	-.02	.48**	.50**						
(12) Life Satisfaction T1	6.25	2.38	1–10	.06**	.15**	.10**	.10**	.08**	.08**	.07**	.01	.47**	.46**	.43**					
(13) General Anxiety T1	1.91	0.86	1–5	-.05*	-.15**	-.04	.04	.05*	-.01	-.02	.05*	-.42**	-.49**	-.45**	-.48**				
(14) Depression T1	2.77	1.22	1–7	-.05*	-.16**	-.08**	-.06**	-.07**	-.01	-.11**	.07**	-.57**	-.53**	-.62**	-.58**	.74**			
(15) Life Satisfaction T2	6.45	2.25	1–10	.10**	.13**	.07*	.08**	.10**	.10**	.04	.00	.41**	.41**	.35**	.60**	-.45**	-.51**		
(16) General Anxiety T2	1.78	0.83	1–5	-.07*	-.12**	-.04	-.03	-.06*	.00	-.04	.05	-.40**	-.44**	-.40**	-.43**	.76**	.67**	-.56**	
(17) Depression T2	2.74	1.21	1–7	-.05	-.13**	-.05	-.02	-.06*	-.01	-.09**	.09**	-.52**	-.46**	-.52**	-.50**	.63**	.76**	-.61**	.77**
