# Supplementary material for: Call me maybe: Risk factors of impaired social contact during the COVID‐19 pandemic and associations with well‐being
Source: Br J Soc Psychol. 2022 May 26:10.1111/bjso.12546. Online ahead of print. doi: 10.1111/bjso.12546 (PMC9348265; doi:10.1111/bjso.12546)
Supplement: Supplementary file 1 — Appendix S1 [file BJSO-9999-0-s001.zip › Data analysis/Analyses conducted with SPSS (Descriptives and Step 01)/Data file/Codebook SPSS Datafile.pdf]

Variable Name	Description	Coding
lfdn	Participant ID	
Dropout	Participation at the second measurement point	0 = no 1 = yes
age_t1	age	Open response
sex_t1	gender	1 = female, 2= male, 3= diverse, 4= prefer not to answer
edu_t1	Highest Education Degree	1 = PhD<br>2 = Completed Master Degree / Magister / Diploma / Staatsexamen<br>3 = Completed Bachelor Degree<br>4 = General qualification for University attendance (Abitur / Fachabitur)<br>5 = Certificate vocational training school<br>6 = Certificate Secondary Education (Realschule)<br>7 = Certificate Secondary Education (Hauptschule)<br>8 = Certificate Polytechnic High School after 8th grade<br>9 = Completed Secondary Education School<br>10 = other degree (e.g. abroad)<br>11 = dropped out of school without a degree<br>12 = did not attend school
hh_member_t1	Number of Household Members	Open Response
hh_partner_t1	Living with Partner	0 = not selected, 1= selected
hh_child_t1	Living with Children	0 = not selected, 1= selected
hh_parents_t1	Living with Parents	0 = not selected, 1= selected
hh_family_t1	Living with other Family Members	0 = not selected, 1= selected
hh_friends_t1	Living with Friends	0 = not selected, 1= selected
hh_colleagues_t1	Living with Colleagues	0 = not selected, 1= selected
hh_students_t1	Living with Students	0 = not selected, 1= selected
hh_member_na_t1	Prefer not to answer	0 = not selected, 1= selected
work_con_t1	Work Environment	1= workplace<br>2 = from home due to the pandemic<br>3= from home as usual<br>4 = could not work due to the pandemic<br>5 = prefer not to answer
si_confreq_t1	Social Contact: Absolute Frequency	1 = not at all<br>2= once per week<br>3 = several times per week<br>4 = once per day<br>5 = several times per day

si_confreq_comp_t1	Social Contact: Relative Frequency	1 = much less contact<br>4 = just as much contact<br>7 = much more contact
si1_conam_t1	Social Contact: Absolute Number	1 = no one<br>2= with one person<br>3 = 2-5 persons<br>4 = 6-20 persons<br>5 = more than 20 persons
si1_conam_comp_t1	Social Contact: Relative Number	1 = contact with far fewer people<br>4 = contact with just as many people<br>7 = contact with far more people
si_con_face_t1	Frequency Face to Face communication	1 = not at all<br>2= once per week<br>3 = several times per week<br>4 = once per day<br>5 = several times per day
si2_con_skype_t1	Frequency Video communication	1 = not at all<br>2= once per week<br>3 = several times per week<br>4 = once per day<br>5 = several times per day
si2_con_phone_t1	Frequency Phone communication	1 = not at all<br>2= once per week<br>3 = several times per week<br>4 = once per day<br>5 = several times per day
si2_con_TM_t1	Frequency Instant Message communication	1 = not at all<br>2= once per week<br>3 = several times per week<br>4 = once per day<br>5 = several times per day
aut_t1	Autonomy	1 = not at all<br>7 = very much
com_t1	Competence	1 = not at all<br>7 = very much
rel_t1	Relatedness	1 = not at all<br>7 = very much
lone_t1	Loneliness	1 = never<br>5 = very often
mood_ang_t1	Mood: anger	1 = very rarely<br>5 = very often
mood_anx_t1	Mood: anxiety	1 = very rarely<br>5 = very often
mood_hap_t1	Mood: happy	1 = very rarely<br>5 = very often
mood_sad_t1	Mood: sad	1 = very rarely<br>5 = very often
mood_dis_t1	Mood: disgust	1 = very rarely

		5 = very often
mood_bor_t1	Mood: bored	1 = very rarely<br>5 = very often
life_sat_t1	Life Satisfaction T1	1 = not satisfied at all<br>10 = completely satisfied
life_sat_t2	Life Satisfaction T2	1 = not satisfied at all<br>10 = completely satisfied
dep_t1	Depression T1	1 = never<br>7 = nearly always
dep_t2	Depression T2	1 = never<br>7 = nearly always
anx_t1	Anxiety T1	1 = not at all<br>7 = very much
anx_t2	Anxiety T2	1 = not at all<br>7 = very much
med_comp_phone_t1	Competence Phone	1 = not competent at all<br>7 = very competent
med_comp_TV_t1	Competence TV	1 = not competent at all<br>7 = very competent
med_comp_radio_t1	Competence Radio	1 = not competent at all<br>7 = very competent
med_comp_game_t1	Comptence Game Consoles	1 = not competent at all<br>7 = very competent
med_comp_PC_t1	Competence PC	1 = not competent at all<br>7 = very competent
med_comp_tablet_t1	Competence Tablet	1 = not competent at all<br>7 = very competent
med_comp_smphone_t1	Competence Smartphone	1 = not competent at all<br>7 = very competent
hex_x	Extraversion	1 = strongly disagree<br>5 = strongly agree
